# Activism for intersectional justice in sport sociology: Using intersectionality in research and in the classroom

**DOI:** 10.3389/fspor.2022.920806

**Published:** 2022-10-18

**Authors:** Emma Calow

**Affiliations:** Bowling Green State University, Bowling Green, OH, United States

**Keywords:** intersectionality, activism in sport, sport sociology, graduate students, athlete activism, methodology, teaching

## Abstract

This perspective paper considers what scholars and teachers of sport sociology can (un)learn by applying the concept of intersectionality in research and in the classroom. I focus on contemporary forms of activism in the context of sport in the United States (U.S.) and demonstrate intersectionality's utility through three examples of athlete activism from the past 10 years led by sports people. Although each example is focused on a particular axis of difference and domination, such as sexual harassment (read: gender) and Black Lives Matter (read: race), I show that the cause at stake is always already intersectional. This has consequences for the field of sport studies/sport sociology; in engaging in intersectional research, sport sociologists and researchers alike can inform policymakers in sport in the decision-making process. In the final part of the paper, I offer insight from my positionality as a graduate student through reflection on how I—and my colleagues—might understand our role within the “matrix of domination” that characterizes both our subject and our field. As novice sport scholars, graduate students can translate the theoretical meanings and purpose of intersectionality into lived reality by being intentional in what and how we teach and research. In this case, I suggest that intersectional justice in sport does not just mean on the track/field/court; it can also mean in the classroom, thereby expanding our notion of what activism “in sport” is and looks like.

## Introduction

Standing on the podium at the summer 2020 Tokyo Olympic Games, U.S. shot-putter Raven Saunders raised her arms into an “X” shape above her head after the silver medal was placed around her neck. Her tacit and peaceful yet resounding protest represented the “intersection where all who are oppressed meet” (1). As a member of the Black and LGBTQIA community, as well as an avid advocate for mental health, Saunders used her “X” to symbolize the cultural, political, and social minoritized positions many are forced to occupy: “For me, just being who I always aspired to be, to be able to be me and not apologize for it…[and] show the younger generation that no matter what they tell you, no matter how many boxes they try to fit you in, you can be you” [(2), para. 6].

Saunders' very peaceful and very public protest represents a generation of fourth wave athlete activists who are deeply cognizant of and attuned to the sociopolitical climate in which they compete (3). Many of this generation of athletes–both professional and collegiate–hold multiple minoritized identities [e.g., (4)]. As a result, athletes speaking out on systemic issues within and/or beyond sport are forced to confront the very issues they seek to eradicate (i.e., sexism, racism, homophobia, etc.). Moments of calls to action by sportspeople in recent years, like Saunders', have reenergized the collective global movement of Black Lives Matter and galvanized conversations across geographical borders about anti-racism, including the ways in which race as a system of classification and difference-making operates. The power intersectionality holds as a concept guiding methodological and analytical studies emerge from its ability to recognize how social and cultural processes of domination and difference-making are mutually constitutive.

Saunders' story as a Black American athlete who identifies as a queer woman is unique and not uncommon. However, the quick dismissal of a story like hers *is* common in academic and non-academic spaces. There lies a myriad of questions (and possible answers) as to what led to Saunders' decision to peacefully protest at this specific moment at this specific event in the way that she did and what it may mean. What can we learn from Saunders' story as an Olympian and her journey to get there in relation to what it means to live “at the intersection”? What barriers did she face and what obstacles did she overcome in sport as a person living “at the intersection”? In what ways can her experiences reveal the *intersecting oppressions* that Black queer women are often forced to confront in a system that privileges whiteness, maleness, and straightness (5–9)?

In the aftermath of first wave feminism that focused exclusively on the needs and wants of white women in the U.S., feminists of color argued that social equity does not mean giving the same to everyone; rather, it means acknowledging the diversity of experiences of women in U.S. society and meeting the individual needs and rights of different groups of women accordingly (10). In other words, a multidimensional way of being and living requires a multidimensional framework of inquiry (6, 11, 12).

Importantly, there is *no hierarchy of oppression* (13). First wave feminism intentionally hierarchized oppression by treating one form of discrimination as more important than other forms. In other words, sexism preceded racism, classism, ableism, etc., thus first wave feminism focused solely on white women's issues. The sexism *and* racism *and* classism *and* ableism, etc., experienced by women of color was consequently discounted. In perhaps the first to do so, Sojourner Truth captured these contradictions in her 1851 speech “Ain't I A Woman?” Here, Truth (14) expounded the nature of her experiences in enslavement as a Black woman, enforcing the realities of racist *and* sexist practices to her majority white audience.

This perspective paper thus considers what sport sociologists can (un)learn by applying intersectionality in research and in the classroom. To demonstrate this, I focus on three examples of athlete activism from the past 3 years led by sportswomen in the U.S. Although each example is focused on a particular axis of difference and domination, my aim is to show that the cause at stake is always already intersectional. This has consequences for how the root cause of social (in)justice is explained by sports sociologists, coupled with how we can encourage sports policymakers to respond. In the final part, I offer insight from my positionality as a graduate student, reflecting on how I and my colleagues might understand our role within the “matrix of domination” that characterizes both our subject and our field. I suggest that intersectional justice in sport does not just mean on the track/field/court; it can also mean in the classroom, thereby expanding our notion of what activism “in sport” is and looks like.

## Intersectionality in sport sociology

Intersectionality's utility rests on dispelling oversimplifications or generalizations of a particular group of people and their lived experiences. Western patriarchal society is built upon various systems that interact to constitute a person's everyday experiences, treatment, and livelihood (i.e., racism, sexism, capitalism, ableism, heterosexism). These systems do not operate independently from one another. Intersectionality is a way to examine such interlocking nature of these systems and the ways in which they may simultaneously converge and/or diverge (11). Black women living in the U.S. experience sexism, racism, etc., often at the same time; the nature of that experience, however, differs among individual Black women (6, 11). That is, systems of oppression are not experienced in the same way. Intersectionality is a “complementary tool to other forms of knowledge for combating white racism, sexism, classism, homophobia, elitism, ageism, xenophobia, ableism, and ethnocentrism in qualitative research practices and paradigms” [(15), p. 14]. In this case, learning about and listening to individuals whose lives are lived at the intersection in sport serves as an integral, productive way to move forward with sport sociological research and teaching for intersectional social justice.

To think intersectionally is thus to think about how lives are impacted through a prism of multiple systems of oppression as they interact with one another to create “effects.” This requires critical thinking about why and how the livelihoods of individuals living at the intersections, like Raven Saunders', are impacted in more ways than one in certain contexts, as well as how and where dynamics of power show up (or do not) (16). To examine the activism of athletes–who and how–and the effects of said activism–legal, social, and/or organizational consequences faced by athlete–is one way to go about facilitating this critical thinking and encouraging intersectional analysis into the classroom by examining the meanings made and sociological implications of such activism. This in turn challenges established ideas of what is known, enables deeper understandings of what is yet to be known, and the impact of such on the knower (i.e., the athletes).

Because sport, culture, and society are deeply intertwined to the point of inseparability [e.g., (17)], sport sociology looks to examine the meaning of sport in/to society, and how sport–including physical culture, including dance and fitness (18), and sport organizations (19)–operates as a tool of socialization, as well as a barrier to inclusion–specifically, the ways through which sport facilitates structures of power^1^ and the impact this has on its participants. Accordingly, examining sport through a sociological lens allows deeper understanding into the relationship between sport, cultural ideology, and politics. More critically, the function of sport in western society depends on who you ask: from a sociological perspective, the operationalization and purpose sport is complex, often enmeshed in larger institutional practices that serve a certain purpose for a certain people. As a critical framework, intersectionality allows for in-depth exploration into why and how power and difference-making operate, and what contexts and categories are unmarked or unaccounted for. Such exploration leads the way to revealing solutions by filling in necessary gaps. Intersectionality's relevance in the study of sport thus lies in its ability to reflect the complexities of living within systems of power that help certain groups of people in some contexts and hurt another group in other contexts since “no one is ever just privileged or oppressed” [(16), p. 133].

When written and presented in a way that is accessible for all (20, 21), sport sociology can and should *add nuanced reality* that informs sport policy and social practice (22). This way of studying sport has the potential to transform how people experience sport and how sport experiences people. As Newman (22) argues:

To add reality is not necessarily to present one's politics as science. Rather, it is to dig deeper and to reveal new associations—to produce new gatherings—which might reveal how old associations are at work in the social world; to reveal the political physics of the hyperobject that is sport (p. 272).

A growing number of studies across a range of topics/issues in sport elicits the in-depth knowledge and meaning making possible when intersectionality is used as methodological and theoretical frameworks [e.g., (23)]. To study sport critically, then, means to examine the inequities it (re)produces and enables as an institution that was historically created by and for white men. Rooted intellectually in Black feminism (11), intersectionality has made major contributions to these kinds of critical studies; what is known and can be known about the role and impact of sport on the experiences of women and minoritized communities is foundational to intersectional research and teaching.

Intersectional research reveals, for example, the impact of race and gender on the representation, opportunity, and experiences of Black women in sport (24–28), the role media plays in constructions of women athletes of color (29), the complexities of sports coaching (30, 31), the homogenizing nature of white feminist scholarship of sport and leisure (32), and stories of athletes with disabilities [e.g., (33)]. Further, a handful of sport sociology research that employs intersectionality is embedded in activism for social justice through its explicit aid in calls to action for inclusive, empowering practice within and beyond sport. For instance, intersectionality's use in youth sports policy and intervention for social inclusion and emancipation (34), intersectionality's purpose in exposing sport as a structure that contributes to racialization and economic injustice (35), and the benefit of intersectional research to understand the enabling factors for women in leadership positions in sport (36).

Utilizing intersectionality as a methodological and analytical framework in research design and practice would benefit the field of sociology of sport in ways that would deepen understanding and broaden knowledge about the interactions of, and contexts around, systems of power and privilege (16). For example, intersectionality could reveal valid and valuable information about not just how and why activism is occurring and by whom, but also the importance of when and where and why of that activism. These contextual elements provide a gateway into discovering the intricate relational, non-hierarchal, and non-linear workings of history, power, and privilege (16). As such, I consider intersectionality as system-centered (16); the interactions of systems of power and privilege that constitute the lives of individuals who are marginalized and privileged are processes that are “fully interactive, historically co-determining, and complex” (p. 129). Systems of power rely on the negative meanings society allocates to and reproduces in articulations of difference (11); to be perceived and thus categorized as different is to be lumped together as the ‘other' to the point of assuming everyone in that group experiences sexism or racism, etc., in the same way. Yet, the “social power in delineating difference need not be the power of domination; instead it can be the source of social empowerment and reconstruction” [(11), p. 1242]. Moreover, grouping people by their social identities can lead to ignoring intragroup differences (11). For this reason, I see intersectionality as less group-centered (which can fetishize the study of “difference”) as it is system-centered. In other words, questioning how and why and by whom “difference” is made, and the impact this difference-making has on individuals on a structural, political, and representational level (11).

Intersectionality's relevance thus lies in its ability to reflect the complexities of living amidst such systems of power, and the operationalization of these systems in terms of helping some individuals in certain contexts and hurting many in other contexts–or “thinking about the way power has clustered around certain categories and is exercised against others” [(11), p. 1297]. Ultimately, employing intersectionality in sport sociology research can expose inequities, the realities of those inequities, and possible solutions that may not be otherwise discoverable.

## Examples of athlete activism that reveal intersectional causes

Activism can be defined as intentional action that challenges the status quo and aims toward positive social change and equity (37). Reflecting the heterogeneity of actions to achieve a certain goal in life, activism is not confined to one way of doing; there are indeed a myriad of ways to ‘do' activism in and through sport. In what follows, I provide three examples of athlete activism from the past 10 years led by sportswomen. Because the lived experiences of all women in sport are always constituted by the systems of power that shape them (5, 7, 38), focusing on sportswomen's activism illuminates the reality that the cause at stake is always intersectional. Again, since “no one is only privilege or only oppressed” [(16), p. 133], stories like these are a way of underlining this complex reality.

### Megan Rapinoe

In September 2016, U.S. national women's soccer player, Megan Rapinoe, took a knee during a pre-game playing of the national anthem in solidarity with Colin Kaepernick, former National Football League player. This very peaceful, very public protest was Rapinoe's nod in support of the Black Lives Matter movement, specifically police brutality against people of color in the U.S. Given the centrality of nationalism in U.S. soccer [e.g., (39)], this form of activism was perceived as unpatriotic through the refusal to conform to American ideals; therefore, Rapinoe's kneeling garnered significant backlash from sports fans online, specifically on social media [e.g., (40)], and from the National Women's Soccer League (USNWSL)–so much so that new polices prohibiting future kneeling were created in the aftermath of Rapinoe's protest.

Rapinoe has never been shy about speaking up for causes she believes in. For example, Rapinoe channeled a vibrant blue, red, and white suit accompanied by an unmissable sparkling red handbag bearing the words, “in gay we trust” at last year's MET Gala. Here, Rapinoe, through creative and personal style, capitalized on her social status as an athlete at a major cultural event to raise awareness for LGQBT+ rights within and beyond sport. She was also a focal leader of the soccer team's case for equal pay to the courts and won (41).

This demonstrates, in part, her recognition of the platform from which she speaks (i.e., professional U.S. sport) and her continual courage in challenging the status quo and the powers that be.

Speaking to her kneeling during the national anthem, Rapinoe reflects on her identity and experience as a gay woman in the U.S.: “I know what it means to look at the flag and not have it protect all of your liberties. It was something small that I could do and something that I plan to keep doing in the future and hopefully spark some meaningful conversation around it” (42). Although Rapinoe was kneeling for Black Lives Matter, the cause of such kneeling stems from the complex realities of living as a person with multiple minoritized identities–in this case, as a gay sportswoman–due to sexism and homonegativism^2^ (9, 43). In other words, Rapinoe's activism is intersectional because the relations between these systems affect her life intersectionally (16). The purpose of her protest was to challenge a single system of difference-making (i.e., racism), yet the cause and consequence of the protest accounts for multiple systems of difference-making and the interaction of such systems (i.e., sexism, homonegativism, capitalism).

In the context of the U.S., Rapinoe's whiteness affords her a privilege that her teammates of color and athletes of color in other sports are not^3^. For instance, in Kaepernick's case, kneeling during the national anthem garnered significant attacks online on his identities as a Black man; conversely for Rapinoe, kneeling called for questions and comments around athletes' role in sport and the larger society (40). Although Kaepernick and Rapinoe were both attacked online, the frequency and content of attack was not the same. In other words, Kaepernick's racial and gender identity was under persistent direct scrutiny, whereas Rapinoe's identities as a white gay sportswoman were not directly attacked. A reason for this may be that whiteness is the unmarked category wherein the “more powerful are defined as the normative standard” [(16), p. 133]. Rapinoe's activism was not perceived as threatening or exasperating as Kaepernick's because, in that moment, racism and white supremacy worked together to create different experiences for and treatment of the athletes. In this context, Rapinoe protested without any real threat to her life, unlike Kaepernick (44). Her intersecting identities mean that different forms of discrimination and privilege are always in process; the interaction of these systems that largely constitute and shape those identities (i.e., racism, sexism, homonegativism) mediate reactions to and meanings of her explicit activism toward a specific cause.

### Hailey Danz

In many cases, visibility and representation can serve as forms of activism wherein someone's presence in sport *is* the protest. U.S. Paralympian Hailey Danz, who won silver in paratriathlon at the Tokyo 2020 Olympics, feels “for the first time in [her] life…proud to be gay” (45). Writing about her story of self-discovery and affirmation, Danz penned an essay on Team USA website:

I know there are a lot of people who say that sexuality has no place in sport; that the press should stop sensationalizing who we love and simply focus on the game. To those people let me say this: it was by seeing openly gay athletes that I've been able to work through my shame and insecurities and accept who I am [(45), para. 16].

Although not a direct challenge to the powers that be nor, like Rapinoe, an action that many may consider a threat to American nationalism, stories like Danz's that are available to the public (and will be for long time on account of the internet) and written by athletes who are marginalized in a multitude of ways demonstrate the power of sport as a site for cultural visibility and representation. The inclusion of voices like Danz's in professional sport paves the way to reimagine the meaning and role of sport in identity formation, identity mediation, and, at times, identity negation (46).

That said, it is no secret that sport is an institution that privileges able-bodiedness [e.g., (47)]. Progress has been made relative to access to and participation in sport for differently-abled bodies. Problems persist, however, including stereotypes around and lack of holistic inclusion and care of disabled athletes in sport and physical activity, which is indicative of wider society's treatment and attitude toward people who have a disability (48, 49). Moreover, the centrality of the empowerment rhetoric by leaders in and organizers of elite disability sport (such as the Paralympics) implies that athletes with cognitive and/or physical disabilities are not empowered anywhere–that they are in constant influx of disempowerment (50).

Danz's explicit activism for LGBQT+ inclusion and rights cannot be divorced from her experiences as a gay female athlete with a physical disability. In this instance, multiples systems of power (i.e., ableism, sexism, and homonegativism) converge and interact with one another to create a process of difference-making. Danz paradoxically uses the very thing that often marginalizes her (i.e., sport) as a platform from which to speak about the ways sport is integral in the sociocultural formation and affirmation of people's identities. Seeing “openly gay athletes” in elite sport as inspirational and using this representation as a tool for self-acceptance infers that seeing straight athletes in elite sport is the norm. Moreover, like Rapinoe, Danz's whiteness is key to her story of activism, including how it is told (i.e., positive narrative framing in sport media) and how it is received (i.e., little to no direct attack on her character and life).

### Naomi Osaka

Before the 2021 French Open began, then-world number two Naomi Osaka revealed to the world her intentional absence from any press before or after matches, citing the lack of consideration for athletes' mental health and her personal “long bouts of depression” (51). Similar to Danz, although this was not an explicit action against a particular social inequity, it *was* an action that challenges the status quo and inadvertently called for meaningful change. In refusing to speak to press, Osaka disrupted the norms of professional sport culture by prioritizing her mental wellbeing. Accordingly, Osaka stood up for herself and athletes like her who so often are told to “shut up and do their job.”

Osaka is not the first to decline press conferences and interviews (52); she is, however, one of the first among a new generation of professional athletes to leverage their sociopolitical power in sport (3). In this case, Osaka unapologetically supported people and causes close to her heart, especially for the communities of which she is a part. For example, during the 2020 U.S. Open amid the COVID-19 pandemic, she wore seven masks for her seven matches, each of which bore the names of unarmed Black Americans murdered by armed law enforcement. This peaceful protest demonstrated Osaka's discursive power as an athlete who holds multiple identities (53).

Osaka's activism for mental health is a by-product of the interactions of various systems of difference-making in sport and the wider society in which she competes, specifically toward Black women (6). Racism and sexism are rife for a Black woman in the U.S.; this is exacerbated in sport culture by constructions and (re)productions of hegemonic white femininity (54–56). That said, Osaka's age, ability, and socio-economic status no doubt warrants privilege in most, if not all contexts; these markers often mean that when Osaka engages in activism–whether direct or indirect–more power is afforded to her than others, and, ultimately, her message is heard (Calow, 2021). The interactions among and across other markers (i.e., race and gender) also mean, however, that her activism is sometimes met with criticism, particularly through media (57).

Moreover, to openly advocate for mental health as a Black sportswoman is to directly challenge common expectations about the role of the athlete in society. The seemingly rebellious act of prioritizing wellbeing over performance and profit–to effectively put oneself at the expense of others' entertainment–is to actively disrupt the dominant cultural narrative that athletes can and should “do it all.” Although the single issue at stake is about professional athletes' mental health, the cause is multi-issued about the livelihoods, treatment, and experiences of Black women in sport and beyond.

## What we can (un)learn from learning about intersectionality

Looking to the examples of athlete activism noted above, using intersectionality as a mode of critical analysis allows a recognition of and points toward the intricate realities and processes of what it can mean to engage in activism for social justice in sport, including the nuanced power dynamics that constitute said activism (58). For example, in Rapinoe's case, in what ways did Rapinoe's whiteness contribute to the reception of her activism for Black Lives Matter? What role do systems of difference-making, such as sexism and homonegativism, play in informing the causes and consequences of Rapinoe's activism? These are the kinds of critically evocative questions that intersectionality brings to the forefront as a methodological and analytical framework. Moreover, these examples of activism can be brought into classroom as part of a pedagogical premise rooted in intersectionality. In Osaka's case, for example, centering her voice and examining her activism valorizes a women of color's experience as a form knowledge-production (59). That is, a key action step teachers can take is asking students to unpack taken-for-granted sporting events/moments that expose the realities of discriminatory systems: What is going here? Who is making the decision and why? Who is impacted and how? In what ways can we address the inequity? What policies would you create/dismantle? Etc.

Choosing to teach, let alone to learn, about intersectionality is not an easy undertaking, especially in a predominantly white institution with majority first-generation students who most likely have never had to think about what it means to be minoritized in a multitude of ways. Like most things in life, to teach and learn about intersectionality is an ongoing, messy process. Nonetheless, the purpose of employing and embodying intersectionality in the classroom is to guide students in their critical understanding of the workings of systems of difference-making, in turn empowering them to become engaged thinkers and doers beyond the classroom in their local and global communities. The choice to undertake the teaching of intersectionality within a field whose history is unidimensional (i.e., the exclusion of non-white voices and the lack of critical attention to the multidimensional issues in sport that affect individuals/athletes of color) in a classroom is in itself a form of activism; this is perhaps the first key action step teachers of sport sociology can take.

As a doctoral candidate, I teach Introduction to Women's Studies, Introduction to Ethnic Studies, and Sport and Social Justice. It is in these classes wherein learning and unlearning moments materialize for students and I. These classes have different content but the same premise: how and why and where power operates in everyday society and culture. It is through these teaching experiences and moments that my understanding about the ways through which intersectionality can be used as a critical thinking tool to approach certain topics and issues deepens. It is in the classroom where I embody intersectional justice through pedagogical practices that in turn informs what and who is the focus of my research and in teaching. For example, including work by and voices of individuals who look different to students into class weekly readings is one practice I have seen is integral to facilitating learning about the purpose of intersectionality.

I think often about the ways in which I contribute to systems of difference-making and discrimination since I largely benefit from them^4^, and in what ways I can disrupt this process. Learning about intersectionality and unlearning what I have been socially and culturally conditioned to believe and think has taught me thus far that I can use my positionality for meaningful change, albeit slow, steady change. As a graduate student whose work is rooted in feminist methodology, part of my task is to translate the complex theoretical meanings and purpose of intersectionality in ways that enable undergraduate students to relate, connect, and understand so as to apply intersectionality as a mode of thinking and/or doing in their own lives through different contexts and experiences. As such, intersectional justice in sport does not just mean on the track/field/court; it can also mean in the classroom, thereby expanding the notion of what activism “in sport” is and looks like.

In research, sport sociology needs intersectionality to fill in necessary gaps of how power operates in certain everyday sporting contexts and culture–gaps that may not be filled otherwise. Sport sociologists can and should be critically attentive to what systems of difference-making are at play in a given context, who is affected (both negatively and positively or both), and how these processes and relationships can be analyzed and in turn unpacked. In so doing, sport sociologists can relay data to policymakers and decision-makers to ensure sport is a more socially just space than it stands today^5^. This is why we need to think and do intersectionally: the kind of work we choose to do on a day-to-day basis as teachers and scholars is imperative to enhancing what we know and do not know about sport from a sociological perspective.

## Conclusion

At the 2019 Pan American Games, U.S. hammer-thrower Gwen Berry raised her fist on the podium during the national anthem, akin to Tommie Smith and John Carlos at the 1968 Mexico City Games. Justifying her fist-raising, Berry claimed that the national anthem never spoke to her or people who look like her (63), placing her in the cannon of athletes who have taken it upon themselves to politicize sport in a public manner through peaceful means. As a result, Berry was banned from competing for a year and fined by the U.S. Olympic and Paralympic Committee (U.S.O.P.C), effectively rendering her a “broke Black woman” (para. 10). Meanwhile, Smith and Carlos' names were inducted into the USOPC Hall of Fame in reward for their activist efforts fifty years prior.

The cause and consequences of Berry's activism underscores why intersectionality belongs in the field of sport sociology. Intersectionality teaches us who is pushed out and why and in what they are pushed out. It legitimizes the need for marginalized voices, but also emphasizes the necessity of a system-centered approached if change is to be attained (16). For example, questioning how oppression manifests and in what contexts. In so doing, sociologists of sport can intervene in scholarship and in the classroom. That is, intersectionality can be used a research lens and has a pedagogical practice. Sport has *never not been political*; the structures of power and systems of difference-making that constitute people's lives have been and are always already embedded within sport as it stands today (64). If we are to change how sport is conceptualized, operationalized, and taught, we must utilize intersectionality to reveal what and where problems persist and how to fix them, including whose voices and knowledges are subjugated and whose are privileged (59). As Crenshaw (11) asserts, “through an awareness of intersectionality, we can better acknowledge and ground the differences among us and negotiate the means by which these differences will find expression in constructing group politics” (p. 1299). Most of all, the use of intersectionality in the study of sport on the premise of social justice is integral if we are to enhance the critical scope and depth of the sport sociology field [e.g., (65)]. To do so is not without risk and will not be easy. But nothing worth doing is ever easy.

## Data availability statement

The original contributions presented in the study are included in the article/supplementary material, further inquiries can be directed to the corresponding author.

## Author contributions

The author confirms being the sole contributor of this work and has approved it for publication.

## Conflict of interest

The author declares that the research was conducted in the absence of any commercial or financial relationships that could be construed as a potential conflict of interest.

## Publisher's note

All claims expressed in this article are solely those of the authors and do not necessarily represent those of their affiliated organizations, or those of the publisher, the editors and the reviewers. Any product that may be evaluated in this article, or claim that may be made by its manufacturer, is not guaranteed or endorsed by the publisher.

## Footnotes

^1^At this time of writing, I conceptualize “system” and “structure” as interchangeable, since both imply formidable scaffolding of organization, categorization, and treatment of individuals and institutions across the U.S. That's not to say they are definitively the same, however. Here, the terms structure/system are used in a connotative, rather than a denotative, sense.

^2^The term homonegativism (9, 43) places more accountability on people than to merely excuse their discriminatory attitudes and behavior. Homo*phobia* implies an irrational fear of people who identify within the LGBQT+ community; homonegativism describes purposeful, not irrational, negative ideologies and behaviors, thus encompassing the social and cultural contexts that facilitate such behaviors.

^3^Race is just one structure of privilege; Rapinoe is also an able-bodied successful athlete, but for this case I focus on race, specifically whiteness, given the issue she is protesting is racism.

^4^I am a white, Northern Irish, straight, able-bodied woman from a middle-class family. Understanding these intersecting identities is to recognize that it is possible to be objective utilizing a critical theory to analyze and teach the importance of intersectionality with vantages of power/privilege.

^5^For example, the International Swimming Federation's most recent ban of transgender women athletes from elite/international competition has created an uproar among sport scholars and sportspeople alike. Emerging research in this area continues to prove the ubiquitous and varied everyday discrimination and systemic barriers trans athletes are forced to confront in their respective sporting spaces [e.g., (60–62)]. This research reveals the complex ways in which systems of difference-making operates and affects individuals living at the intersections, thereby pointing to why thinking and doing research in a multi-dimensional way matters.
